# Classification and Error Estimation for Discrete Data

**DOI:** 10.2174/138920209789208228

**Published:** 2009-11

**Authors:** Ulisses M Braga-Neto

**Affiliations:** Department of Electrical and Computer Engineering, Texas A&M University, College Station, TX 77845, USA

**Keywords:** Genomics, classification, error estimation, discrete histogram rule, sampling distribution, resubstitution, leave-one-out, ensemble methods, coefficient of determination.

## Abstract

Discrete classification is common in Genomic Signal Processing applications, in particular in classification of discretized gene expression data, and in discrete gene expression prediction and the inference of boolean genomic regulatory networks. Once a discrete classifier is obtained from sample data, its performance must be evaluated through its classification error. In practice, error estimation methods must then be employed to obtain reliable estimates of the classification error based on the available data. Both classifier design and error estimation are complicated, in the case of Genomics, by the prevalence of small-sample data sets in such applications. This paper presents a broad review of the methodology of classification and error estimation for discrete data, in the context of Genomics, focusing on the study of performance in small sample scenarios, as well as asymptotic behavior.

## 1. INTRODUCTION

In high-throughput Genomics applications, the objective is often to classify different phenotypes based on a panel of gene expression biomarkers, or to infer underlying gene regulatory networks from gene expression data. It is often advantageous to discretize gene expression data, for data efficiency and classification accuracy reasons. Classification of discrete data is a subject with a long history in Pattern Recognition [1-7]. In Genomics applications, this methodology has been applied both in classification of discretized gene expression data [8, 9], and in discrete gene expression prediction and the inference of boolean genomic regulatory networks, *via* the *binary coefficient of determination* (CoD) [10-12].

The most often employed discrete classification rule is the *discrete histogram rule* [1, 3, 4, 6, 13]. This classification rule has many desirable properties. For example, it can be shown that it is strongly universally consistent, meaning that, regardless of the particular distribution of the data, this rule can eventually learn the optimal classifier from the data, as the sample size increases, with probability one. In addition, the discrete histogram rule is simple enough to allow the exact analytical study of many of its properties.

Once a classifier is obtained from sample data, its performance must be evaluated. The most important criterion for performance is the *classification error*, which is the probability of making an erroneous classification on a future sample. The classification error can be computed exactly only if the underlying distribution of the data is known, which is almost never the case in practice. Robust *error estimation* methods must then be employed to obtain reliable estimates of the classification error based on the available data. An error estimator is a sample-based statistic, the bias and variance (and thus the root mean square error, RMS) properties of which determine how consistently the error estimator is near the true classification error, considering all possible sample training data sets from a given population. More generally, all statistical questions regarding the accuracy of the error estimator can be answered through the joint sampling distribution of the error estimator and true probability of error [14]. From an epistemological perspective, error estimation has to do with the fundamental question of the validity of scientific knowledge [15]. The quality of the error estimate determines the accuracy of the predictions that can be performed by the inferred model, and thus its scientific content.

Both classifier design and error estimation are complicated, in the case of Genomics, by the prevalence of *small-sample* data sets in such applications. With a small training sample set, the designed classifier will be, on average, more dissimilar to the optimal classifier, and thus have a larger classification error. In addition to that, in a small-sample setting, one must use the same data to both design the classifier and assess its validity, which requires data-efficient error estimators, and this in turn calls for careful study of performance.

It is the goal of the present paper to present a broad review of the methodology of classification and error estimation for discrete data, in the context of Genomics. The paper is organized as follows. Section 2 illustrates the application of discrete classification in Genomics through a pair of simple examples. Section 3 formalizes the problem, with particular emphasis on the discrete histogram classification rule. Section 4 reviews the most common error estimators used in discrete classification, commenting briefly on their properties. Sections 5 through 7 contain the bulk of the literature review on the subject. Section 5 reviews results on the small-sample performance of discrete classification; these are analyses that must hold for a given finite number of samples. This section reviews exact and approximate expressions for performance metrics of the actual and estimated errors for the discrete histogram rule; complete enumeration methods that can deal with intractable cases such as conditional performance metrics; distribution-free results on small-sample performance, with emphasis on the pioneering work of G.F. Hughes; as well as recent analytical results that indicate that ensemble classification methods may be largely ineffective in discrete classification. Section 6, by contrast, focuses on the large-sample performance of discrete classification; this is a more technical section, which reviews asymptotic results on whether optimal performance is reached, and how fast, as the sample size increases. Finally, Section 7 reviews the binary coefficient of determination (CoD).

## 2. DISCRETE CLASSIFICATION IN GENOMICS

The objective of *classification* is to employ a set of *training data*, consisting of independently observed known cases, and obtain a fixed rule to classify, as accurately as possible, unknown cases from the same population. The training data consists of carefully measured values of predictive variables and a response variable for each case. The response variable in classification is always *discrete*, i.e., it assumes a finite number of values; in fact, it is often binary, indicating one of two states, such as distinct cell phenotypes, disease severity, and so on.

If the predictor variables are also discrete, then one is in the context of *discrete classification*, also known as *multinomial classification *[13] and *discrete discriminant analysis* [7]. Additionally, in Statistics, the term *categorical data analysis* is often employed to refer to the statistical analysis of discrete data [16]. In Genomics, the predictor variables often correspond to the expression of a set of suitably selected genes; for discrete classification, gene expression must first be discretized into a finite number of intervals --- methods to accomplish this are described in [8, 9]. Note that the finite values taken on by the discrete predictors could be numeric (e.g., the mid-point value of an expression range), or purely categorical, as is often done by alluding to “up-regulated” and “down-regulated” genes. This distinction is immaterial in the case of the most commonly used discrete classification rule, known as the *discrete histogram rule *[1, 3, 4, 6, 13]. The discrete histogram rule simply assigns to each combination of possible values of the predictor variables a response value that is decided by majority voting among the observed response values. As will be seen in this paper, this simple rule has many desirable and interesting properties.

Fig. (**1**) depicts an example of how the discrete histogram rule would function in the case of cell phenotype classification based on the discretized expression values of two genes. Classification is between the phenotypes of “treated” and “untreated” cells (e.g., presence or absence of some drug in the culture, presence of enough nutrients *vs*. starvation, normal *vs*. abnormal cells, and a host of other possible conditions), and gene expression is discretized in ternary values, corresponding to down-regulated, basal, and up-regulated values. There are therefore 3^2^ = 9 possible combinations of observable values, or “bins”, which can be organized in this case into a 3×3 matrix. In this example, the observed training data set contains a total of 40 cases, with an equal number of cases in each of the “treated” and “untreated” categories (sometimes called a “balanced experimental design” in Statistics). The *counts* of observed response values over each of the bins are shown in the figure. The majority class is underlined in each case, and this would be the class assigned to a future case by the discrete histogram rule if the observed gene expression values fall into that particular bin. Note that there are two particular cases that require attention: there could be a tie between the counts over a bin, and no values might be observed in the training data over a bin (missing data). These cases can be resolved by randomly picking one of the classes or, if one wants to avoid random classifiers, one can break ties, in a fixed manner, in favor of one of the classes; for example, one might classify such cases as “untreated”. Based on the resulting discrete classifier in this particular example, one might posit that up-regulation of both genes is associated with treatment of the cells.

Fig. (**2**) depicts another example, which illustrates how the discrete histogram rule would be applied in the case of discrete gene expression prediction; this constitutes the basic building block for the inference of gene regulatory networks [10, 11]. Gene expression values have been discretized into binary values, indicating activation or not of the particular gene, and the expression of three genes (the predictor variables) is used to predict the expression of a fourth gene (the response, or *target*, variable). Note that the number of bins in this case is 2^3^ = 8 . The bins are represented side by side in Fig. (**2**), rather than organized into a matrix as in   Fig. (**1**). This clearly makes no difference to the discrete histogram rule (an important point we will return to in the next Section). Note that the values of all variables (predictors and target) are coded into 0 and 1, and that ties in this example are broken in favor of the class 1, that is, high-expression. As can be seen, prediction is based on a total of 40 cases, i.e., 40 instances of the 4-tuple consisting of the three predictor genes and the target gene. Note that the values 0 and 1 for the target gene are not represented equally, so the design is “unbalanced.” It will be rarely the case in gene prediction that the design is balanced, since here one cannot possibly or meaningfully specify in advance the target classes for the observations; this is a very important difference with respect to the previous case of phenotype classification, where it is often possible and meaningful to do so.

The validity of any scientific conclusions made based on the previous classification models depends, naturally, on the accuracy of the obtained classifiers. In addition, it critically depends on the reliable *estimation* of said accuracy, based on the available data. These issues will be approached in the sequel.

## 3. DISCRETE CLASSIFICATION RULES

Examples of discrete classification rules include the discrete histogram rule, mentioned in the previous section, as well as the maximum-mean-accuracy rule of [5], and many other examples of discrete rules used in Data Mining [17]. Among these, the discrete histogram rule is by far the most used one in practice. The discrete histogram rule is “natural” for categorical problems, not only due to its simplicity as majority voting over the bins, but also because it corresponds to the plug-in rule for approximating the optimal Bayes classifier, as we discuss below. In this section, we formalize the problem of discrete classification, which allows us to examine the properties of the discrete histogram rule, including classification accuracy and its estimation from data.

**Fig. (1):**
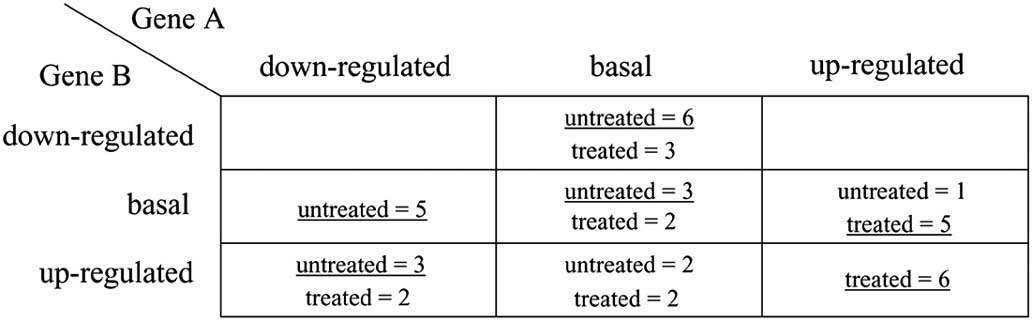
Phenotype classification based on discrete gene expression.

Let the predictor variables be denoted by *X*_1_, *X*_2_,..., *X_d_*, and the response variable be denoted by *Y*. We assume here, for simplicity, that $Y\in \left\{0,1\right\}$ is a binary response variable. In discrete classification, each *X_i_* is allowed to take on a finite number *b_i_* of values. The *feature space* is thus finite, consisting of $b=\Pi_{i=1}^{d}b_{i}$ possible states (see the matrix in Fig. **1**). As remarked in connection with Fig. (**2**), for the discrete histogram rule, the space can be reorganized in any way one likes. Therefore, we adopt a (bijective) mapping between the original feature space and the sequence of integers 1,...,*b*, and may equivalently assume, without loss of generality, a single predictor variable *X* taking on values in the set {1,...,*b*}. The value *b* is the total number of bins into which the data are categorized --- this parameter provides a direct measure of the complexity of discrete classification.

The properties of the discrete classification problem are completely determined by the (discrete) joint probability distribution between the predictor *X* and the target *Y*:

PX=i,Y=j, for i=1,...,b and j=0,1.

Given the identity $P\left(X=i,Y=j\right)=P\left(X=i\left|Y=j\right.\right)P\left(Y=j\right),$ it becomes clear that the discrete classification problem is determined by 2*b*+2 positive parameters *c*_0_ = *P*(*Y*=0), *c*_1_ = *P*(*Y*=1), and $p_{i}=P\left(X=i\left|Y=0\right.\right),q_{i}=P\left(X=i\left|Y=1\right.\right),$ for *i* = 1,...,*b*. Note that the parameters are not independent, since one must have $c_{0}+c_{1}=1,\sum p_{i}=1,\mathrm{and}\;\sum q_{i}=1.$

Through Bayes' theorem, these model parameters determine the posterior probabilities $P\left(Y=j\left|X=i\right.\right)$ for the classification problem, $P\left(Y=0\left|X=i\right.\right)=\frac{P\left(Y=0,X=i\right)}{P\left(X=i\right)}=\frac{c_{0}p_{i}}{c_{0}p_{i}+c_{1}q_{i}}$ with $P\left(Y=1\left|X=i\right.\right)=1-P\left(Y=0\left|X=i\right.\right)$. Therefore, the classifier Ψ^*^ that achieves the minimum *probability of misclassification* $P\left(Y\neq \Psi \left(X\right)\right),$ known as the *Bayes classifier* [13], is given by

(1) $$\psi^{*}\left(X=i\right)=\left\{\begin{array}{c} 1,\;P\left(Y=0\left|X=i\right.\right)<P\left(Y=1\left|X=i\right.\right)\\ 0,\;P\left(Y=0\left|X=i\right.\right)\geq P\left(Y=1\left|X=i\right.\right) \end{array}\right\}=\left\{\begin{array}{c} 1,\;c_{0}p_{i}<c_{1}q_{i}\\ 0,\;c_{0}p_{i}\geq c_{1}q_{i} \end{array}\right.$$

It can be shown that if there are two or more discrete features in the original feature space (such as in Fig. **1**), and these features are independent conditionally to *Y*, i.e., within each class, then the Bayes classifier Ψ^*^ is a linear function of those features [13, p.466].

The minimum probability of misclassification, or *Bayes error*, achieved by the Bayes classifier, can be written as

(2) $$\varepsilon^{*}=\sum_{i=1}^{b}P\left(X=i,Y=1-\psi^{*}\left(X=i\right)\right)=\sum_{i=1}^{b}\left[c_{0}p_{i}I_{c_{1}q_{i}>c_{0}p_{i}}+c_{1}q_{i}I_{c_{0}p_{i}\geq c_{1}q_{i}}\right]=\sum_{i=1}^{b}\min\left\{c_{0}p_{i},c_{1}q_{i}\right\}.$$

Here, *I_A_* is an *indicator variable*, which is 1 when condition *A* happens, and 0, otherwise. Since $\sum \min\left\{a_{i},b_{i}\right\}\leq \min\left\{\sum a_{i},\sum b_{i}\right\},$ it follows that $0\leq \varepsilon^{*}\leq \min\left\{c_{0},c_{1}\right\}.$ The upper bound is reached if (though not only if) *p_i_* = *q_i_*, for all *i* = 1,...,*b*. The largest Bayes error possible is 0.5, which is achieved if and only if *c*_0_ = *c*_1_ = 0.5 and *p_i_* = *q_i_*, for all *i* = 1,...,*b* (total confusion between the classes).

The Bayes error is a measure of distance between the classes, and it provides a lower bound on classification performance. For discrete histogram classification, the predictor variables in the original feature space should be chosen so that the Bayes error is as small as possible.

In practice, one almost never knows the model parameters completely, and therefore one does not know the Bayes classifier. One must rely instead on designing a classifier from sample *training data*; one hopes that such a sample-based classifier is close in some sense to the Bayes classifier. The classifier produced by the discrete histogram rule becomes indeed very close to the Bayes classifier, as sample size increases, in a few important senses; this will be discussed in Section 6.

Given sample data $S_{n}=\left\{\left(X_{1},Y_{1}\right),...\left(X_{n},Y_{n}\right)\right\}$ containing *n* independent and identically distributed (i.i.d.) samples, one defines the *bin counts* *U_i_*,*V_i_* as the observed number of points with *X* = *i* for class 0 and 1, respectively, for *i* = 1,...,*b*. For example, in Fig. (**2**), the bin counts *U_i_* are 4,1,3,3,2,4,0,4, while the bin counts *V_i_* are 2,3,3,4,0,3,3,1. The *discrete histogram classification rule* is given by majority voting between the bin counts over each bin:

(3) $$\psi_{n}\left(S_{n},X=i\right)=I_{U_{i}<V_{i}}=\left\{\begin{array}{c} 1,\;U_{i}<V_{i}\\ 0,U_{i}\geq V_{i} \end{array},\;i=1,...,\right.b.$$

When a specific training sample *S_n_* = *s_n_* is given, then the values of the bin counts *U_i_* and *V_i_* become fixed, leading to a fixed *designed* discrete histogram classifier $\psi_{n}\left(\cdot \right)=\psi_{n}\left(S_{n}=s_{n},.\right).$ For an example, see Fig. (**2**). Note that, in the above definition, as in Fig. (**2**), we choose to break ties in favor of class 0.

Ordinarily, the samples in *S_n_* are drawn form a mixture of the two class populations, and therefore the numbers N0=∑Ui and N1=∑Vi  of samples in classes 0 and 1, respectively, are random variables. In this *full sampling* case, *N*_0_ and *N*_1_ are binomially distributed: *N*_0_ : *Binomial*(*n,c*_0_) and *N*_1_ : *Binomial*(*n,c*_1_), with *N*_0_+*N*_1_=*n*. In addition, the vector of bin counts (*U*_1_,...,*U_b_*,*V*_1_,...,*V_b_*) is jointly multinomially distributed, with parameters (*n,c*_0_*p*_1_, ...,*c*_0_*p_b_*,*c*_1_*q*_1_,...,*c*_1_*q_b_*) ; it follows that the individual bin counts are binomially distributed: *U_i_* : *Binomial*(*n,c*_0_*p_i_* and *V_i_* : *Binomial*(*n,c*_1_*q_i_*, for *i* = 1,...,*b*. On the other hand, one may design the experiment in such a way that the number of samples *N*_0_ = *n*_0_ and *N*_1_ = *n*_1_ are fixed in advance, with *n*_0_+*n*_1_ = *n*, and the class populations are sampled separately. To avoid bias, the values of *n*_0_ and *n*_1_ should be chosen to reflect the a priori probabilities *c*_0_ and *c*_1_ of each class: *n*_0_=[*c*_0_*n*] and *n*_1_[*c*_1_*n*], where [*x*] denotes the nearest integer to *x*. In this *stratified sampling* case, the vector of bin counts (*U*_1_,...,*U_b_*) is multinomially distributed with parameters (*n*_0_,*p*_1_,...,*p_b_*), and is independent from the vector of bin counts (*V*_1_,...,*V_b_*), which is multinomially distributed with parameters (*n*_1_,*q*_1_,...,*q_b_*); the individual bin counts are still binomially distributed: *U*_i_ : *Binomial*(*n*_0_,*p_i_*) and *V*_i_ : *Binomial*(*n*_1_,*q_i_*), for *i* = 1,...,*b*.

The discrete histogram rule is the “plug-in” rule for discrete classification, that is, if one plugs the standard maximum-likelihood (ML) estimators of the unknown model parameters *c*_0_,*c*_1_ and {*p_i_*},{*q_i_*},

(4) c0ˆ=∑iUin,c1ˆ=∑iVin and piˆ=Ui∑iUi,qiˆ=Vi∑iVi,for i=1,...,b,

in the expression for the Bayes classifier in (1), one obtains precisely the histogram classifier in (3). Since the standard ML estimators in (4) are consistent, meaning that they converge to the true values of the parameters as the sample size increases, one would expect the discrete histogram classifier to approach the optimal Bayes classifier as more samples are acquired, which is indeed the case; we come back to this issue in Section 6.

The most important performance criterion for the designed classifier *Ψ_n_* is its accuracy on independent (e.g., future) data, which are assumed to come from the same population as the given training data. This accuracy is measured by the probability of misclassification $\varepsilon_{n}=P\left(Y\neq \psi_{n}\left(X\right)\right),$, where (*X,Y*) is i.i.d. with all (*X_i_,Y_i_*) in *S_n_*. This is known as the *classification error*. It is clear that

(5) $$\varepsilon_{n}=\sum_{i=1}^{b}P\left(X=i,Y=1-\psi_{n}\left(X=i\right)\right)=\sum_{i=1}^{b}\left[c_{0}p_{i}I_{V_{i}>U_{i}}+c_{1}q_{i}I_{U_{i}\geq V_{i}}\right]$$

Being a function of the random variables *U_i_* and *V_i_*, *ε_n_* is a random variable (*ε_n_* ceases to be random, becoming fixed, when a fixed training data set *S_n_*, and thus fixed values of *U_i_* and *V_i_*, are specified). The expected value of *E*[*ε_n_*] over the training data *S_n_* has an important meaning in the context of classification rules. It does not depend on a particular set of training samples, but it gives the average classification error over all possible training data; therefore it is an intrinsic performance measure of the classification rule for the particular problem (i.e., joint distribution of *X* and *Y*) and sample size *n*.

## 4. ERROR ESTIMATION FOR DISCRETE CLASSIFICATION

In practice, the underlying probability model is unknown, and the classification error *ε_n_* has to be estimated from the sample data using an *error estimator* $\hat{\varepsilon_{n}}$. An error estimator is a function of the classification rule Ψ_n_ and the sample data *S_n_*. Therefore, it is a random variable through dependency on the random training data *S_n_*. If the error estimator depends on any additional random factors, sometimes called *internal factors*, it is called *randomized*, otherwise it is said to be *nonrandomized*. Examples of the latter include the *apparent error* or *resubstitution* [18], and *leave-one-out* [19] error estimators, whereas popular examples of randomized error estimators include cross-validation [19-22] and all bootstrap-based error estimators [23-25].

As the classification error *ε_n_* itself, a nonrandomized error estimator $\hat{\varepsilon_{n}}$ produces a fixed value once the training data set *S_n_* is specified (“running the estimator again” on the data never alters the result), which is not the case for a randomized error estimator. Internal random factors introduce *internal variance* that adds to the *total variance* of an error estimator, which measures how dispersed its estimates can be for varying training data from the same population. Note that the internal variance is zero for nonrandomized estimators. Randomized estimators typically reduce the unwanted extra internal variance through averaging based on intensive computation. See [26, 27] for a detailed discussion of issues regarding randomized and non-randomized error estimators, and internal and full variance.

The variance of the error estimator, by itself, does not address its relationship to the quantity to be estimated, namely, the actual classification error. Relevant performance metrics that do so are discussed next. The *bias* $E\left[\hat{\varepsilon_{n}}-\varepsilon_{n}\right]$ of an error estimator measures whether, on average, it overestimates the true error, or underestimates it, whereas the *deviation variance* $\mathrm{Var}\left(\hat{\varepsilon_{n}}-\varepsilon_{n}\right)$ measures the spread of the deviation between estimated and actual errors; it can in fact be written as

(6) $$\mathrm{Var}\left(\hat{\varepsilon_{n}}-\varepsilon_{n}\right)=\mathrm{Var}\left(\hat{\varepsilon_{n}}\right)+\mathrm{Var}\left(\varepsilon_{n}\right)-2\rho \left(\hat{\varepsilon_{n}},\varepsilon_{n}\right)\sqrt{\mathrm{Var}\left(\hat{\varepsilon_{n}}\right)\mathrm{Var}\left(\varepsilon_{n}\right)}$$

a remarkable formula that combines the variances of the actual error and error estimator, and their *correlation* $\rho \left(\hat{\varepsilon_{n}},\varepsilon_{n}\right).$. Small bias is of small significance if the deviation variance is large; this would mean that on average the error estimator is close to the true error, but that in fact the estimate for any particular sample set is likely to be far away from the true error. The *root mean-square error* (RMS)

(7) $$R\mathrm{MS}\left(\hat{\varepsilon_{n}}\right)=\sqrt{E\left[{\left(\hat{\varepsilon_{n}}-\varepsilon_{n}\right)}^{2}\right]}=\sqrt{E{\left[\hat{\varepsilon_{n}}-\varepsilon_{n}\right]}^{2}+\mathrm{Var}\left(\hat{\varepsilon_{n}}-\varepsilon_{n}\right)}$$

conveniently combines both the bias and the deviation variance into a single measure, and is widely adopted for comparison of error estimator performance. Additional performance measures include the *tail probabilities* Pεnˆ−εn>τ, for τ>0, which concern the likelihood of outliers, as well as the consistency of the error estimator; the *conditional bias* $E\left[\hat{\varepsilon_{n}}-\varepsilon_{n}\left|\hat{\varepsilon_{n}}\right.\right]=\hat{\varepsilon_{n}}-E\left[\varepsilon_{n}\left|\hat{\varepsilon_{n}}\right.\right]$ (resp. conditional deviation variance and RMS); and *confidence intervals* [*a,b*] such that $P\left(a\leq \varepsilon_{n}\leq b\left|\hat{\varepsilon_{n}}\right.\right)>1-\alpha ,$ for 0 ≤ *α* ≤1, which give bounds on the true error corresponding to a given precision *α*, the observed error estimate, and the sample size. Confidence intervals express statistical power in error estimation --- more powerful methods will produce shorter confidence intervals for the true error at the same sample size. A very important fact is that all of the aforementioned performance metrics, and in fact any others, can be determined if one has knowledge of the *joint sampling distribution* of the vector of actual and estimated errors $\left(\varepsilon_{n},\hat{\varepsilon_{n}}\right)$. Section 5 reviews exact analytical methods to compute these performance metrics, as well as complete enumeration methods that allow the computation of the joint sampling distribution of actual and estimated errors.

The resubstitution error estimator $\hat{\varepsilon_{n}^{r}}$ [18] is the simplest data-efficient alternative; it is simply the apparent error, or the proportion of errors the designed classifier makes on the training data itself. Clearly,

(8) $$\hat{\varepsilon_{n}^{r}}=\frac{1}{n}\sum_{i=1}^{b}\min\left\{U_{i},V_{i}\right\}=\frac{1}{n}\sum_{i=1}^{b}\left[U_{i}I_{V_{i}>U_{i}}+V_{i}I_{U_{i}\geq V_{i}}\right].$$

For example, in Fig. (**2**), the resubstitution estimate for the classification error is 12/40 = 0.3. It is easy to see that plugging the ML estimators of the model parameters in (4) into the formula for the Bayes error (2), results in expression (8). Therefore, resubstitution for the discrete histogram rule is the plug-in estimator of the Bayes error in discrete classification. The resubstitution estimator is clearly nonrandomized, and it is very fast to compute. This estimator is however always optimistically biased in the case of the discrete histogram rule, in the sense that $E\left[\hat{\varepsilon_{n}^{r}}\right]\leq E\left[\varepsilon_{n}\right],$ for any sample size and distribution of the data. In fact, it can be shown that

(9) $$E\left[\hat{\varepsilon_{n}^{r}}\right]\leq \varepsilon^{*}\leq E\left[\varepsilon_{n}\right]$$

so that the average resubstitution estimate bounds from below even the Bayes error; this fact seems to have been demonstrated for the first time in [1] (see also [2]). Observe though that this is not guaranteed to apply to any individual training data and classifier, but only to the average over all possible training data. The optimistic bias of resubstitution tends to be larger when the number of bins is large compared to the sample size; in other words, there is more overfitting of the classifier to the training data in such cases. On the other hand, resubstitution tends to have small variance. In cases where the bias is not too large, this makes resubstitution a very competitive option as an error estimator. In fact, the next Section contains results that show that resubstitution can have smaller RMS than even complex error estimators such as the bootstrap, provided that sample size is large compared to number of bins. In addition, it can be shown that as the sample size increases, both the bias and variance of resubstitution vanish (see Section 6). Finally, it is important to emphasize that these observations hold for the discrete histogram rule; for example, the resubstitution estimator is not necessarily optimistically-biased for other (continuous or discrete) classification rules.

The leave-one-out error error estimator ${\hat{\varepsilon }}_{n}^{l}$ [19] removes the optimistic bias from resubstitution by counting errors committed by *n* classifiers, each designed on *n*-1 points and tested on the remaining left-out point, and dividing the total count by *n*. A little reflection shows that

(10) $$\hat{\varepsilon_{n}^{l}}=\frac{1}{n}\sum_{i=1}^{b}\left[U_{i}I_{V_{i}\geq U_{i}}+V_{i}I_{U_{i}\geq V_{i}-1}\right].$$

For example, in Fig. (**2**), the leave-one-out estimate for the classification error is 15/40 = 0.375. This is higher than the resubstitution estimate of 0.3. In fact, by comparing (8) and (10), one can see that, in all cases, it is true that ${\hat{\varepsilon }}_{n}^{l}\geq {\hat{\varepsilon }}_{n}^{r}$ . In particular, $E[{\hat{\varepsilon }}_{n}^{l}]\geq E[{\hat{\varepsilon }}_{n}^{r}]$ showing that the leave-one-out estimator must be necessarily less optimistic than the resubstitution estimator. In fact, it is a general result (not restricted to discrete histogram classification), that $E[{\hat{\varepsilon }}_{n}^{l}]=E\left[\varepsilon_{n-1}\right]$ making this estimator almost unbiased. As it turns out, this bias reduction is accomplished at the expense of an increase in variance [26]. The leave-one-out estimator is however nonrandomized.

A randomized estimator is obtained by selecting randomly *k* *folds* of size *n-n/k* , counting the errors committed by *k* classifiers, each designed on one of the folds and tested on the remaining points not in the fold, and dividing the total count by *n*. This yields the well-known *k*-fold *cross-validation* estimator [19-22]. The process can be repeated several times and the results averaged, in order to reduce the internal variance associated with the random choice of folds. The leave-one-out estimator is a cross-validation estimator with *k* = *n*; therefore, cross-validation is not randomized in this special case (it is also nonrandomized for other choices of *k* if one considers all possible folds of size *n-n/k*, which can be a very large number if *n* is large or *k* is small). It is a general result (not restricted to discrete histogram classification) that the *k*-fold cross-validation estimator ${\hat{\varepsilon }}_{n}^{\mathrm{cvk}}$ is such that $E\left[{\hat{\varepsilon }}_{n}^{\mathrm{cvk}}\right]=E\left[\varepsilon_{n-n/k}\right].$

Another class of popular randomized error estimators are based on the the idea of bootstrap [23-25]. A “bootstrap sample” consists of *n* equally-likely draws with replacement from the original training data. The basic bootstrap estimator $\hat{\varepsilon_{0}}$ is similar to cross-validation, in that it counts the errors committed by *B* classifiers, each designed on a bootstrap sample and tested on training points not in the bootstrap sample, and divides the count by the total number of test points (which is variable). The number *B* of bootstrap samples must be made large to reduce the internal variance associated with bootstrap sampling (the ideal case *B* = ∞ leading to a nonrandomized estimator; in practice, this would be achieved by a very large, but finite, *B*, which is equal to the number of all possible draws of *n* indices with replacement from the index set 1,...,*n*). The estimator $\hat{\varepsilon_{0}}$ tends to be pessimistically biased, and therefore a convex combination with resubstitution, which is optimistically biased (in the case of discrete histogram classification), was proposed in [24]:

(11) $${\hat{\varepsilon }}_{n}^{\mathrm{b632}}=\left(1-0.632\right){\hat{\varepsilon }}_{r}+0.632{\hat{\varepsilon }}_{0}$$

This is known as the **0.632** *bootstrap* error estimator, and is quite popular in Machine Learning applications [17]. It has small variance, but can be very slow to compute. In addition, it will fail when the resubstitution estimator is too optimistic. A variant called the *0.632+ bootstrap* error estimator was introduced in [25], in an attempt to correct this problem. All cross-validation and bootstrap error estimators tend to be computationally intensive, due to the large number of classifier design steps involved and the need to reduce internal variance by averaging over a large number of iterations.

## 5. SMALL-SAMPLE PERFORMANCE OF DISCRETE CLASSIFICATION

The fact that the distribution of the vectors of bin counts (*U_1_*,...,*U_b_*) and (*V_1_*,...,*V_b_*) is multinomial (see Section 3), and thus easily computable, along with the simplicity and parallel among equations (2), (5), (8), and (10), for the Bayes error, actual error, resubstitution error, and leave-one-out error, respectively, allow the detailed analytical study of the small-sample performance of the discrete histogram classification rule and the associated resubstitution and leave-one-out error estimators.

### 5.1. Analytical Study of Actual Classification Error

From (5) it follows that the expected error over the sample is given by

(12) $$E\left[\varepsilon_{n}\right]=\sum_{i=1}^{b}\left[c_{0}p_{i}E\left[I_{V_{i}>U_{i}}\right]+c_{1}q_{i}E\left[I_{U_{i}\geq V_{i}}\right]\right]=\sum_{i=1}^{b}\left[c_{0}p_{i}P\left(V_{i}>U_{i}\right)+c_{1}q_{i}P\left(U_{i}\geq V_{i}\right)\right]=c_{1}+\sum_{i=1}^{b}\left(c_{0}p_{i}-c_{1}q_{i}\right)P\left(V_{i}>U_{i}\right).$$

The computation of the probability *P*(*V_i_*>*U_i_*) depends on whether full or stratified sampling is used. In the full sampling case, the pair (*U_i_*,*V_i_*) has a *trinomial* joint distribution with parameters (*n*,*c_0_p_i_*,*c_1_q_i_*), so that

(13) $$P\left(V_{i}>U_{i}\right)=\sum_{l>k}\left(\begin{array}{c} n\\ k,l,n-k-l \end{array}\right){\left(c_{0}p_{i}\right)}^{k}{\left(c_{1}q_{i}\right)}^{l}{\left(1-c_{0}p_{i}-c_{1}q_{i}\right)}^{n-k-l},$$

whereas in the stratified sampling case, *U_i_* is independent of *V_i_*, and each is binomially distributed with parameters (*n*_0_,*p_i_*) and (*n*_1_,*q_i_*), respectively, so that

(14) $$P\left(V_{i}>U_{i}\right)=\sum_{l>k}\left(\begin{array}{c} n_{0}\\ k \end{array}\right)p_{i}^{k}{\left(1-p_{i}\right)}^{n_{0}-k}\left(\begin{array}{c} n_{1}\\ l \end{array}\right)q_{i}^{l}{\left(1-q_{i}\right)}^{n_{1}-l}.$$

To obtain the variance $\mathrm{Var}\left[\varepsilon_{n}\right]=E\left[\varepsilon_{n}^{2}\right]-{\left(E\left[\varepsilon_{n}\right]\right)}^{2}$ one needs the second moment $E\left[\varepsilon_{n}^{2}\right]$:

(15) $$E\left[\varepsilon_{n}^{2}\right]=\sum_{i=1}^{b}\left\{c_{0}^{2}p_{i}^{2}P\left(V_{i}>U_{i}\right)+c_{1}^{2}q_{i}^{2}P\left(U_{i}\geq V_{i}\right)\right\}+\sum_{i,j=1i\neq j}^{b}\left\{c_{0}^{2}p_{i}p_{j}P\left(V_{i}>U_{i},V_{j}>U_{j}\right)+c_{0}c_{1}\left[p_{i}q_{j}P\left(V_{i}>U_{i},U_{j}\geq V_{j}\right)+p_{j}q_{i}P\left(U_{i}\geq V_{i},V_{j}>U_{j}\right)\right]+c_{1}^{2}q_{i}q_{j}P\left(U_{i}\geq V_{i},U_{j}\geq V_{j}\right)\right\}$$

This expression involves second-order bin probabilities, e.g., *P*(*V_i_*>*U_i_*,*V_j_*>*U_j_*), which can be computed in a similar fashion to the first-order bin probability in (13) and (14), by using the fact that, in the full sampling case, the vector (*U_i_*,*V_i_*,*U_j_*,*V_j_*) has a multinomial joint distribution with parameters (*n*,*c*_0_*p_i_*,*c*_1_*q_i_*,*c*_0_*p_j_*,*c*_1_*q_j_*), whereas in the stratified sampling case, the vector (*U_i_*,*U_j_*) is independent of the vector (*V_i_*,*V_j_*), and each is trinomially distributed with parameters (*n*_0_,*p_i_*,*p_j_*) and (*n*_1_,*q_i_*,*q_j_*), respectively.

However, computation of the expression in (15) becomes difficult when *n* or *b* are large. But if one can assume that the event {*V_i_* > *U_i_*} is approximately independent of the event {*V_j_* > *U_j_*}, then it can be shown after some algebraic manipulation that cancellations occur in the expression (15), leading to a very simple expression for the variance, which involves only first-order bin probabilities:

(16) $$\mathrm{Var}\left[\varepsilon_{n}\right]=\sum_{i=1}^{b}{\left(c_{0}p_{i}+c_{1}q_{i}\right)}^{2}P\left(V_{i}>U_{i}\right)\left(1-P\left(V_{i}>U_{i}\right)\right)$$

It is proved in [28] that, under a mild distributional assumption, the expression in (16) is asymptotically exact as the number of bins grows to infinity, for fixed sample size.

Fig. (**3**) illustrates the application of the formulas above in an example where stratified sampling is assumed, with *c*_0_ = *c*_1_ = 0.5 (so that, in particular, *n*_0_ = *n*_1_ = *n/*2), and probabilities *p_i_* and *q_i_* given by a parametric Zipf (power-law) model:  *p_i_* = *Ki*^-α^ and *q_i_* = *p_b-i+1_*, for *i* = 1,...,*b*. Here, *K* is a normalizing constant given by $K={\left[\sum_{i=1}^{b}i^{-\alpha }\right]}^{-1}.$. The parameter *α* controls the Bayes error of the model, and is set in all cases to $\alpha =\sqrt{2}.$ We can see in Fig. (**3**) that the expected classification error decreases with increasing sample size as expected. The expected classification error also decreases with increasing bin size, but it starts to increase again after *b* > 16 for *n* = 20. This is an example of the “peaking phenomenon” that affects the expected classification error (see Section 5.4). As for the variance, one can see that it also decreases with increasing sample size, as expected. Except for the anomalous case *b* = 20, the variance seems to be insensitive to bin size. One can also appreciate that the approximation to the variance given by (16) is quite accurate, particularly at larger sample sizes. The good accuracy of the approximation is obtained at a huge savings in computation time. As an example, for *b* = 16 and *n* = 60, it takes more than 30 minutes and less than 1 second to compute the exact and approximate expressions for the variance, respectively, using state-of-the-art computing technology.

### 5.2. Analytical Study of Error Estimators

Similar exact expressions can be derived for the expectation and variance of the resubstitution and leave-out-error estimators, as well as their correlation with the actual error; see [29, 30]. These exact expressions allow one to compute exactly the bias, deviation variance, and RMS of both resubstitution and leave-one-out. This is illustrated in Fig. (**4**), where results for resubstitution (resub), leave-one-out (loo), 10-repeated 4-fold cross-validation (cv), and the .632 bootstrap (b632) are displayed. In this figure, “standard deviation” refers to the square root of the deviation variance. For the 0.632 error estimator, *B* = 100 bootstrap samples are employed. Performance measures for resubstitution and leave-one-out are exact; they are computed using the exact expressions mentioned previously. For the other error estimators, performance measures are derived from a Monte-Carlo computation using 20,000 samples from each probability model. The model is the Zipf parametric model mentioned previously, with *c_0_* = *c_1_* = 0.5, and parameter *α* adjusted to yield $E\left[\varepsilon_{n}\right]\approx 0.20,$ which corresponds to intermediate classification difficulty. Stratified sampling is assumed, with *n* = 20 (so that *n*_0_ = *n*_1_ = 10). The value of *n* was chosen to emphasize small-sample effects. The results show that resubstitution is the most optimistically biased estimator, with bias that increases with complexity, but it is also much less variable than all other estimators, including the bootstrap ones. The cross-validation estimators are the most variable, but are nearly unbiased. The bootstrap estimator is affected by the bias of resubstitution when complexity is high, since it incorporates the resubstitution estimate in its computation, but it is clearly superior to the cross-validation estimators in RMS. Perhaps the most remarkable observation is that, for very low complexity classifiers (around b=4), the simple resubstitution estimator becomes more accurate than the cross-validation error estimators, and as accurate as the 0.632 bootstrap error estimator, according to RMS, despite the fact that resubstitution is typically much faster to compute that those other error estimators (in some cases considered in [26], hundreds of times faster). In our experiments, we observed that this is true for small sample sizes (*n* < 30), low complexity, and low to moderate expected classification errors. This has an important consequence for the inference of genomic boolean regulatory networks: if the number of boolean predictors for a particular gene is small (on the order of 2 or 3), then it is more advantageous to use resubstitution to estimate prediction accuracy than more complicated error estimation schemes.

Analytical exact expressions for the correlation between actual and estimated errors can also be derived [30]. This is illustrated in Fig. (**5**), where the correlation for resubstitution and leave-one-out error estimators is plotted versus sample size, for different bin sizes. In this example, we assume full sampling and the Zipf parametric model mentioned previously, with *c*_0_ = *c*_1_ = 0.5 and parameter *α* adjusted to yield two cases: easy (Bayes error = 0.1) and difficult (Bayes error = 0.4) classification.

We can observe that the correlation is generally low (below 0.3). We can also observe that at small sample sizes, correlation for resubstitution is larger than for leave-one-out cross-validation, and, with a larger difficulty of classification, this is true even at moderate sample sizes. Correlation generally decreases with increasing bin size; in one striking case, the correlation for leave-one-out becomes negative, at the critical small-sample situation of *n* = 20 and *b* = 32. This behavior of the correlation for leave-one-out mirrors the behavior of deviation variance of this error estimator, which is known to be large under complex models and small sample sizes [13, 26, 31], and is in accord with (6).

### 5.3. Complete enumeration methods

As mentioned previously, all the performance metrics of interest for the actual error *ε_n_* and any given error estimator $\hat{\varepsilon_{n}}$ can be derived from joint sampling distribution of the pair of random variables $\left(\varepsilon_{n},\hat{\varepsilon_{n}}\right).$ These include conditional metrics, such as the conditional expected actual error given the estimated error and confidence bounds on the actual error conditioned on the estimated error, which are very difficult to study *via* the analytical approach used in the previous subsections, due to the complexity of the expressions involved.

However, due to the finiteness of the discrete problem, it turns out that the joint sampling distribution of actual and estimated errors in the discrete case can be computed exactly by means *complete enumeration*. Such methods have been extensively used in categorical data analysis [16, 32-35]; complete enumeration has been particularly useful in the computation of exact distributions and critical regions for tests based on contingency tables, as in the case of the well-known Fisher exact test and the chi-square approximate test [32, 33].

Basically, complete enumeration relies on intensive computational power to list all possible configurations of the discrete data and their probabilities, and from this exact statistical properties of the methods of interest are obtained. The availability of efficient algorithms to enumerate all possible cases on fast computers has made possible the use of complete enumeration in an increasingly wider variety of settings.

In the case of discrete classification, recall that the random sample is specified by the vector of bin counts *W_n_* = (*U*_1_,...,*U_b_*,*V*_1_,...,*V_b_*) defined previously, so that we can write *ε_n_* = *ε_n_*(*W_n_*) and $\hat{\varepsilon_{n}}=\hat{\varepsilon_{n}}\left(W_{n}\right).$ The random vector *W_n_* is discrete, and so the random variables *ε_n_* and $\hat{\varepsilon_{n}}$ are also discrete, and so is the configuration space *D_n_* of all possible distinct sample values *w_n_* = (*u*_1_,...,*u_b_*,*v*_1_,...,*v_b_*) that can be taken on by *W_n_*. The discrete joint probability distribution of $\left(\varepsilon_{n},\hat{\varepsilon_{n}}\right)$ is given by:

(17) $$P\left(\varepsilon_{n}=k,\hat{\varepsilon_{n}}=l\right)=\sum_{w_{n}\in D_{n}}I_{\left\{\varepsilon_{n}\left(w_{n}\right)=k,\hat{\varepsilon_{n}}\left(w_{n}\right)=l\right\}}P\left(W_{n}=w_{n}\right),$$

where *P*(*W_n_* = *w_n_*), is a multinomial probability that is computed according to the parameters (*n*,*c*_0_*p*_1_,...,*c*_0_*p_b_*,*c*_1_*q*_1_,...,*c*_1_*q_b_*) as

(18) $$P\left(W_{n}=w_{n}\right)=\left(\begin{array}{c} n\\ u_{1},...,u_{b},v_{1},...,v_{b} \end{array}\right)c_{0}^{\sum_{i}u_{i}}c_{1}^{\sum_{i}v_{i}}\overset{b}{\underset{i=1}{\Pi }}p_{i}^{u_{i}}q_{i}^{v_{i}}$$

Even though the configuration space *D_n_* is finite, it quickly becomes huge with increasing sample size *n* and bin size *b*. In [29] an algorithm is given to traverse *D_n_* efficiently, which leads to reasonable computational times to evaluate the joint sampling distribution when *n* and *b* are not too large. Fig. (**6**) displays the joint distribution $P\left(\varepsilon_{n}=k,\hat{\varepsilon_{n}}=l\right)$ for the resubstitution and leave-one-out cross-validation error estimators, for a small-sample case, *n* = 20 and *b* = 8, and a Zipf probability model of intermediate difficulty (Bayes error = 0.2). One can observe that the joint distribution for resubstitution is much more compact than for leave-one-out cross-validation, which explains in part its larger correlation in small-sample cases.

This approach can be easily modified to compute the conditional sampling distribution $P\left(\varepsilon_{n}=k,\left|\hat{\varepsilon_{n}}=l\right.\right).$ This was done in [14] in order to find exact conditional metrics of performance for resubstitution and leave-one-out error estimators. Those included the conditional expectation $E\left(\varepsilon_{n}\left|\hat{\varepsilon_{n}}\right.\right)$ and conditional variance $\text{V}\mathrm{ar}\left(\varepsilon_{n}\left|\hat{\varepsilon_{n}}\right.\right)$ of the actual error given the estimated error, as well as the 100(1-*α*)% upper confidence bound *γ_α_*, such that $P\left(\varepsilon_{n}<\gamma_{\alpha }\left|\hat{\varepsilon_{n}}\right.\right)=1-\alpha .$

This is illustrated in Fig. (**7**), where the aforementioned conditional metrics of performance for resubstitution and leave-one-out are plotted versus conditioning estimated errors, for different bin sizes. In this example, we assume stratified sampling and the Zipf parametric model mentioned previously, with *c*_0_ = *c*_1_ = 0.5 and parameter *α* adjusted to yield $E\left[\varepsilon_{n}\right]\approx 0.25,$ which corresponds to intermediate classification difficulty. Sample size is fixed at *n* = 20 to emphasize small-sample effects and two bin sizes are considered, *b* = 4,8. The curves for the conditional expectation rise with the estimated error; they also exhibit the property that the conditional expected actual error is larger than the estimated error for small estimated errors and smaller than the estimated error for large estimated errors. A point to be noted is the flatness of the leave-one-out curves. This reflects the high variance of the leave-one-out estimator. Note that the 95% upper confidence bounds are nondecreasing with respect to increasing estimated error, as expected. The flat spots observed in the bounds result from the discreteness of the estimation rule (this phenomenon is more pronounced when the number of bins is smaller).

### 5.4. Distribution-Free Analysis of Performance

Note that the model parameters *p_i_* and *q_i_* must be nonnegative and satisfy the constraints $\sum_{i=1}^{b-1}p_{i}\leq 1$ and $\sum_{i=1}^{b-1}q_{i}\leq 1$. Each of these equations determines a *simplex* *S_b-1_* in (*b*-1)-dimensional Euclidean space. Therefore, given the value of *c*_0_ = *P*(*Y* = 0) (so that *c*_1_=1-*c*_0_ is also known), the discrete classification problem is completely determined by a vector of 2(*b*-1) values, which must be a point in the *model space* $\Pi \left(c_{0}\right)=S_{b-1}\times S_{b-1}$.

In [4], G.F. Hughes provided exact expressions that allow the computation of the average Bayes error $\overset{-}{\varepsilon^{*}}\left(b,c_{0}\right)$ and the average expected actual error $\bar{E\left[\varepsilon \right]}\left(n,b,c_{0}\right)$ for the discrete histogram rule, both averaged over the model space ∏(*c*_0_), by assuming that all models in ∏(*c*_0_) are equally-likely to occur. This provides a distribution-free analysis of performance, some of the qualitative features of which are still valid in particular distributional settings. For example, one of the famous conclusions derived in [4] is that, with *n* and *c*_0_ fixed, the curve of the expected actual *accuracy* $1-\bar{E\left[\varepsilon \right]}\left(n,b,c_{0}\right)$ as a function of number of bins *b* *peaks* around an optimal value b^*^, which increases with increasing sample size *n*. Even though this result was derived in terms of the average accuracy over the model space, and for the discrete histogram rule, this “peaking phenomenon” is in fact observed for the majority of individual distributions, and indeed for the majority of classification rules, both discrete and continuous [36].

Using the expressions in Hughes' paper, we plotted the average Bayes accuracy $1-\overset{-}{\varepsilon^{*}}\left(b,c_{0}\right)$ and average expected actual accuracy $1-\bar{E\left[\varepsilon \right]}\left(n,b,c_{0}\right)$, both as a function of *b*, for various values of *n*, assuming the balanced case *c*_0_ = 0.5; the results are displayed in Fig. (**8**). The curves are plotted as a function of *p* = log_2_*b*. This corresponds to the case where *p* binary predictors are used in the original feature space; for example, the point *p* = 5 in the plot corresponds to 5 binary features, with *b* = 2^5^ = 32. One can easily observe the “peaking phenomenon” in this plot. The optimal number of features moves to the right with increasing sample size *n*, and, regardless of the value of *n*, accuracy tends to the no-information value of 0.5 as the number of predictors increases. Sample size computations can be performed based on the curves of Fig. (**8**); for example, if one has *p* = 3 binary predictors, so that *b* = 8, then sample size should be equal to *n* = 60 at a minimum, according to this analysis. The expressions for these curves are quite complicated and computationally intensive for large *n*; however for small *n*, the expressions become quite simple. For example, with *n* = 2,

$1-\bar{E\left[\varepsilon \right]}\left(2,b,0.5\right)=\frac{1}{2}+\frac{1\;b-1}{2b(b+1)}$

so that the accuracy margin over the no-information value of 0.5 vanishes as 1/*b*. This implies that the decrease is exponential in *p* = log_2_(*b*), as can be gleaned from Fig. (**8**).

Note that peaking ceases to occur as *n* → ∞, which corresponds to the Bayes accuracy (see the next Section). This must be the case, since the Bayes accuracy is known to be nondecreasing in the number of features. The expression for the average Bayes accuracy in the case *c*_0_ = 0.5 is simple; as shown in [4], this is given by 

$1-\overset{-}{\varepsilon^{*}}\left(b,0.5\right)=\frac{3b-2}{4b-2}$

with an asymptotic value (as *b* → ∞) of 0.75 (it is shown in [4] that, for general *c*_0_, this asymptotic value is equal to 1-*c*_0_(1-*c*_0_)). This relatively small value highlights the conservative character of Hughes' distribution-free approach; for example, in practice, where one deals with a fixed distribution of the data, the optimal number of features would typically be larger than the ones observed in Fig. (**8**), so that sample size recommendations based on this analysis tend to be pessimistic --- a fact that was pointed out in [37]. Nevertheless, the qualitative behavior of the analysis is entirely correct. Finally, we remark that Fig. (**8**) closely matches Fig. (**3**) in [4], but larger values of *b* are shown here, which possibly were difficult to compute in 1968.

### 5.5. Performance of Ensemble Methods in Discrete Classification

In [38], Braga-Neto and Dougherty carried out an analysis of the performance of ensemble classification methods [39, 40] when applied to the discrete histogram rule, which provided evidence that such ensemble methods may be largely ineffective in discrete classification. Part of the analysis is similar to the work of Hughes', discussed in the previous subsection, in the sense that it examines the average performance over the model space, assuming equally-likely models. Two methods were considered, namely, the *jackknife* and *bagging* ensemble classification rules obtained from the discrete histogram rule. Briefly, ensemble methods are based on perturbing the training data, designing an ensemble of classifiers based on the perturbed data sets using a given base classification rule (in this case, the discrete histogram rule), and aggregating the individual decisions to obtain the final classifier. Data perturbation is often accomplished by resampling methods such as the jackknife [41] and bootstrap [23] --- the latter case being known as “bagging” [40] --- whereas aggregation is done by means of majority voting among the individual classifier decisions. For the jackknife majority-vote classification rule, it was shown in [38] that, under full sampling and equally-likely classes, the best gain in performance (i.e., decrease in expected classification error) over all models in the model space ∏(*c*_0_) is smaller than the worst deficit (i.e., increase in expected classification error). Any discrepancy in performance however disappears as sample size increases; in particular the following bound is shown to hold:

(19) $$\left|E\left[\varepsilon_{n}^{J}\right]-E\left[\varepsilon_{n}\right]\right|\leq \frac{1}{\mathrm{ne}}\sqrt{\frac{2}{\pi \left(n+1\right)}}$$

where $E\left[\varepsilon_{n}^{J}\right]$ and $E\left[\varepsilon_{n}\right]$ are the expected classification errors of the jackknife and base classification rules, respectively. In addition, an exact expression is given for the average $\left|\bar{E\left[\varepsilon_{n}^{J}\right]}-\bar{E\left[\varepsilon_{n}\right]}\right|$ over the model space ∏(*c*_0_), assuming equally-likely distributions as in the work of Hughes. In the case of equally-likely classes (*c*_0_ = 0.5), the result simplifies to show that the average difference is positive, that is, there is an average deficit (which in fact is shown to still hold if the classes are only approximately equally likely, in a precise sense). The left plot in Fig. (**9**) displays these quantities plotted as a function of sample size, for *p* = 2 (*b* = 4 discrete cells), and for the balanced case, *c*_0_ = 0.5. We can observe in the plot that the best gain (inf) is smaller than the worst deficit (sup) and that there is an average deficit (positive average deviation). The values of inf and sup are actually independent of *b*.

Regarding the bagging case, it is shown in [38] that, given the training data, and for any sample size, number of cells, or distribution of the data, the random bagging classifier converges to the original discrete histogram classifier with probability 1 as the number of classifiers in the ensemble *m* increases, and, furthermore, it also gives the following exponential bound on the absolute difference $\left|\varepsilon_{n,m}^{B}-\varepsilon_{n}\right|$ between the generalization errors of the bagging and the base classifiers,

(20) $$\left|\varepsilon_{n,m}^{B}-\varepsilon_{n}\right|\leq e^{{-2\mathrm{mc}}^{2}}$$

where the constant *c* > 0 does not depend on *m*, but depends in a simple way on the distribution of the data. The difference therefore converges exponentially fast to zero as the number of classifiers in the ensemble increases (for fixed *n*). From this it follows that the difference between expected errors over all training data also converges exponentially fast to zero (the constant *c* is larger, guaranteeing faster convergence, if the classes are more separated, in a precise sense). The right plot in Fig. (**9**) displays the expected error for the bagged discrete histogram classification rule as a function of number of classifiers in the ensemble, for model parameters derived from an actual data set, corresponding to *p* = 2 binary features picked from the SPECT Heart data set of the UCI Machine Learning Repository. The expected classification error for the bagging classifier is found by means of a Monte-Carlo computation using 100,000 simulated training sets, assuming full sampling. The Monte-Carlo computation introduces the wobble visible in the plots (even at this very large number of simulated training sets). Also indicated are the exact expected errors of the base discrete histogram classification rule, by means of dashed horizontal lines. We can see that in all cases bagging leads to a larger expected classification error than the base classification rule, although the deviation quickly converges to zero in each case, in agreement with equation (20) above.

## 6. LARGE-SAMPLE PERFORMANCE OF DISCRETE CLASSIFICATION

Large-sample analysis of performance has to do with behavior of classification error and error estimators as sample size increases without bound, i.e., as *n* → ∞. From a practical perspective, one expects performance to improve, and eventually reach an optimum, as more time and cost is devoted to obtaining an increasingly large number of samples. It turns out that not only this is true for the discrete histogram rule, but also it is possible in several cases to obtain fast (exponential) rates of convergence. Critical results in this area are due to Cochran and Hopkins [1], Glick [6, 42], and Devroye, Gyorfi and Lugosi [13]. We will review briefly these results in this Section.

Recall the bin counts *U_i_* and *V_i_* introduced in Section 3. By a straightforward application of the Strong Law of Large Numbers (SLLN) [43], we obtain that *U_i_/n* → *c*_0_*p_i_* and *V_i_/n* → *c*_1_*q_i_* as *n* → ∞, with probability 1. From this and eqs. (1) and (3), it follows immediately that

(21) $$\lim_{n\to \infty }\psi_{n}\left(X=i\right)=\lim_{n\to \infty }I_{U_{i}<V_{i}}=I_{c_{0}p_{i}<c_{1}q_{i}}=\psi^{*}\left(X=i\right)$$

with probability 1.

that is, the discrete histogram classifier designed from sample data converges to the optimal classifier over each bin, with probability 1. This is a distribution-free result, so it is true regardless of the joint distribution of predictors *X* and target *Y*, as the SLLN itself is distribution-free. One says then that the discrete histogram rule is *universally strongly consistent* [13].

The exact same argument, in connection with eqs. (2), (5) and (8), shows that

(22) limn→∞εn=limn→∞εˆnr=ε∗ with probability 1.

so that the classification error, and also the apparent error, converge to the optimal Bayes error as sample size increases. From the previous equation it also follows that

(23) $$\lim_{n\to \infty }E\left[\varepsilon_{n}\right]=\lim_{n\to \infty }E\left[{\hat{\varepsilon }}_{n}^{r}\right]=\varepsilon^{*},$$

In particular, $\lim_{n\to \infty }E\left[{\hat{\varepsilon }}_{n}^{r}-\varepsilon_{n}\right]=0$ and the bias of resubstitution vanishes with increasing sample size. Recalling (9), one always has $E\left[{\hat{\varepsilon }}_{n}^{r}\right]\leq \varepsilon^{*}\leq E\left[\varepsilon_{n}\right]$ , so that (23) in fact implies that $E\left[\varepsilon_{n}\right]↓\varepsilon^{*}$ , while $E\left[{\hat{\varepsilon }}_{n}^{r}\right]↑\varepsilon^{*}$ , as *n* → ∞.

These results are all based on the SLLN (and are thus distribution-free). The question arises as to the speed with which the limits are attained, as the SLLN can yield notoriously slow rates of convergence. This is not only a theoretical question, as the usefulness in practice of such results may depend on how large a sample size needs to be to guarantee that the discrete classifier or error estimator is close enough to optimality. The answer is that exponential rates of convergence can be obtained, if one is willing to drop the distribution-free requirement. Otherwise, polynomial rates of convergence can be established. These results are briefly reviewed below.

Regarding the discrete histogram rule, with a proviso that ties in bin counts are assigned a class randomly (with equal probability), it is shown in [6, Theorem A], that the following exponential bound on the convergence of $E\left[\varepsilon_{n}\right]$ to ε^*^ applies

(24) $$E\left[\varepsilon_{n}\right]-\varepsilon^{*}\leq \left(\frac{1}{2}-\varepsilon^{*}\right)e^{-\mathrm{cn}},$$

where the constant *c* > 0 is distribution-dependent: 

$c=\log\frac{1}{1-\min_{\left\{i:c_{0}p_{i}\neq c_{1}q_{i}\right\}}{\left|\sqrt{c_{0}p_{i}}-\sqrt{c_{1}q_{i}}\right|}^{2}}$

Interestingly, the number of bins does not figure in this bound. The speed of convergence of the bound is determined by the minimum (nonzero) difference between the probabilities *c*_0_*p_i_* and *c*_1_*q_i_* over any one cell. The larger this difference is, the larger *c* is, and the faster convergence is. Conversely, the presence of a single cell where these probabilities are close slows down convergence of the bound.

On the other hand, a distribution-free bound is provided by [13, Theorem 27.1]:

(25) $$E\left[\varepsilon_{n}\right]-\varepsilon^{*}\leq \sqrt{\frac{b}{2\left(n+1\right)}}+\frac{b}{\mathrm{en}}\leq 1.075\sqrt{\frac{b}{n}}$$

This polynomial *O*(*n*^-1/2^) bound is inferior to the exponential bound in (24), but it does guarantee a fixed rate of convergence that is independent of the distribution.

Regarding convergence of $E\left[{\hat{\varepsilon }}_{n}^{r}\right]$ to ε^*^, and again assuming random tie-breaking over cells, it is shown in [6, Theorem B], that the following exponential bound applies

(26) $$\varepsilon^{*}-E\left[{\hat{\varepsilon }}_{n}^{r}\right]\leq \frac{1}{2}{\mathrm{bn}}^{-1/2}e^{-\mathrm{cn}},$$

*provided that* there is no cell over which *c*_0_*p_i_* = *c*_1_*q_i_*. Here, the constant *c* > 0 is the same as in (24). The presence of a cell where *c*_0_*p_i_* = *c*_1_*q_i_* invalidates the bound in (26) and slows down convergence; in fact, it is shown in [6] that in such a case $\varepsilon^{*}-E\left[{\hat{\varepsilon }}_{n}^{r}\right]$ has both upper and lower bounds that are *O*(*n*^-1/2^), so that convergence *cannot* be exponential. Finally, observe that the bounds in (24) and (26) can be combined to bound the *bias* of resubstitution $E\left[{\hat{\varepsilon }}_{n}^{r}-\varepsilon_{n}\right]$ . We can conclude, for example, that in case there are no cells over which *c*_0_*p_i_* = *c*_1_*q_i_*, convergence of the bias to zero is exponentially fast.

The previous results on the discrete histogram rule concern expectation and bias. In [13], (distribution-free) results on variance and RMS are also given, both for resubstitution and leave-one-out (here, the convention we have adopted of breaking ties in the direction of class 0 is again in effect). For the resubstitution error estimator, one has the following bounds [13, Theorem 23.3]:

(27) $$\mathrm{Var}\left[{\hat{\varepsilon }}_{n}^{r}\right]\leq \frac{1}{n}$$

and

(28) $$R\mathrm{MS}\left({\hat{\varepsilon }}_{n}^{r}\right)\leq \sqrt{\frac{6b}{n}}$$

In particular, both quantities converge to zero as sample size increases. For the leave-one-out error estimator, one has the following bound [13, Theorem 24.7]:

(29) $$R\mathrm{MS}\left({\hat{\varepsilon }}_{n}^{l}\right)\leq \sqrt{\frac{1+6/e}{n}+\frac{6}{\sqrt{\pi \left(n-1\right)}}}$$

This guarantees, in particular, convergence to zero as sample size increases.

An important factor in the comparison of the resubstitution and leave-one-out error estimators for discrete histogram classification resides in the different speeds of convergence of the RMS to zero. Convergence of the RMS bound for the resubstitution estimator is *O*(*n*^-1/2^) (for fixed *b*), whereas convergence of the RMS bound for the leave-one-out estimator is *O*(*n*^-1/4^), thus slower. Now, as remarked in [13, p.419], it can be shown that for some distributions there is also a *lower bound* of kind *O*(*n*^-1/4^) on the RMS of leave-one-out. Therefore, in the worst case, the RMS of leave-one-out to zero is guaranteed to decrease as *n*^-1/4^, and therefore is certain to decrease slower than the RMS of resubstitution. Note that the bad RMS of leave-one-out is due almost entirely to its large variance, typical of the cross-validation approach, since this estimator is essentially unbiased.

## 7. BINARY COEFFICIENT OF DETERMINATION (COD)

In classical regression analysis, the *coefficient of determination* (CoD) gives the relative decrease in unexplained variability when entering a variable *X* into the regression of the dependent variable *Y*, in comparison with the total unexplained variability when entering no variables:

(30) $$\mathrm{CoD}\left(X,Y\right)=\frac{{\mathrm{SS}}_{Y}-{\mathrm{SS}}_{\mathrm{XY}}}{{\mathrm{SS}}_{Y}}$$

where *SS*_*Y*_ and *SS*_*XY*_ are the sums of squared errors associated with entering no variables and entering variable *X* to predict *Y*, respectively. The term *SS*_*Y*_ is proportional to the total variance $\sigma_{Y}^{2}$, which is the error around the mean *µ*_*Y*_ (so that entering no variables in the regression corresponds to using the mean as the predictor).

In classification, a very similar concept was introduced in [10]:

(31) $$\mathrm{CoD}\left(X,Y\right)=\frac{\varepsilon_{Y}^{*}-\varepsilon_{\mathrm{XY}}^{*}}{\varepsilon_{Y}^{*}},$$

where $\varepsilon_{Y}^{*}=\min\left\{P\left(Y=0\right),P\left(Y=1\right)\right\}$ is the Bayes error in the absence of any features, and $\varepsilon_{\mathrm{XY}}^{*}$ is the Bayes error when using feature vector *X* to predict *Y*. By convention, one assumes 0/0 = 1 in the above definition. This *binary coefficient of determination* measures the relative decrease in prediction error of a target variable when using predictor variables, relative to using no predictor variables; notice the remarkable similarity between (30) and (31).

The binary CoD was perhaps the first predictive paradigm utilized in the context of microarray data, the goal being to provide a measure of nonlinear interaction among genes [10]. Even though the binary CoD, as defined in (31), has general application in classification, it has been extensively used in the case of discrete classification and prediction, particularly in problems dealing with gene expression quantized into discrete levels [8, 44] --- see the examples given in Section 2 --- and its use in the inference of gene regulatory networks [11, 12]. As its classic counterpart, the binary CoD is a goodness-of-fit statistic that can be used to assess the relationship between predictor and target variables (e.g., how tight the association between a set of predictor genes and a target gene is).

Even though the definition above employs Bayes errors, the CoD can be likewise defined in terms of the classification error of predictors designed from sample data, using for example the discrete histogram rule. In addition, the actual classification errors will typically need to be computed through error estimation techniques; e.g., one may speak of resubstitution and leave-one-out CoD estimates. All the issues discussed in previous sections regarding classification and error estimation for discrete data generally apply here.

A recent paper [45] defined and studied the concept of *intrinsically multivariate predictive* (IMP) genes using the binary CoD. Briefly, IMP genes are those the expression of which cannot be predicted well by any subset of binary predicting gene expressions, but is predicted very well by the entire set. In [45], the properties of IMP genes were characterized analytically, and it was shown that high-predictive power, small covariance among predictors, a large entropy of the joint probability distribution of predictors, and certain logics, such as XOR in the 2-predictor case, are factors that favor the appearance of IMP. In addition, quantized gene-expression microarray data were employed to show that the gene DUSP1, which exhibits control over a central, process-integrating signaling pathway, exhibits IMP behavior, thereby providing preliminary evidence that IMP can be used as a criterion for discovery of *canalizing* genes, i.e., master genes that constrain (“canalize”) large gene-expression pathways [46].

## 8. CONCLUSION

The importance of discrete classification in Genomics lies in its broad application in problems of phenotype classification based on panels of gene-expression biomarkers and inference of gene regulatory networks from gene-expression data, where data discretization is often employed for data efficiency and classification accuracy reasons. This paper presented a broad review of methods of classification and error estimation for discrete data, focusing for the most part on the discrete histogram rule, which is the classification rule most employed in practice for discrete data, due to its excellent properties, such as low complexity and small data requirement (under small number of cells), and universal consistency. The most important criterion for performance is the classification error, which can be computed exactly only if the underlying distribution of the data is known. In practice, robust error estimation methods must be employed to obtain reliable estimates of the classification error based on available sample data. This paper reviewed analytical and empirical results concerning the performance of discrete classifiers (in terms of the classification error) as well as of error estimators for discrete classification. Those results were categorized into small-sample results --- small-sample data being prevalent in Genomics applications --- and large-sample (i.e., asymptotic) results. The binary Coefficient of Determination was also reviewed briefly; it provides a measure of nonlinear interaction among genes and is therefore very useful in the inference of gene regulatory networks. Progress in classification and error estimation for discrete data, particularly the analysis of performance in small-sample cases, has a clear potential to lead to genuine advances in Genomics and Medicine, and therefore the study of such methods is a topic of considerable research interest at present.

## Figures and Tables

**Fig. (2):**
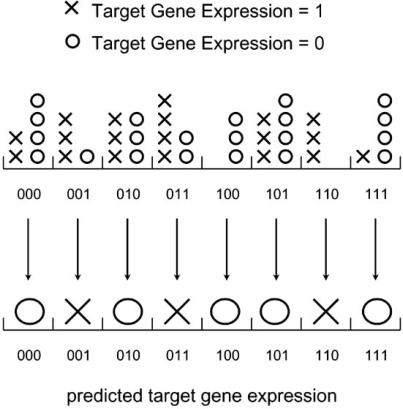
Prediction of discrete gene expression.

**Fig. (3):**
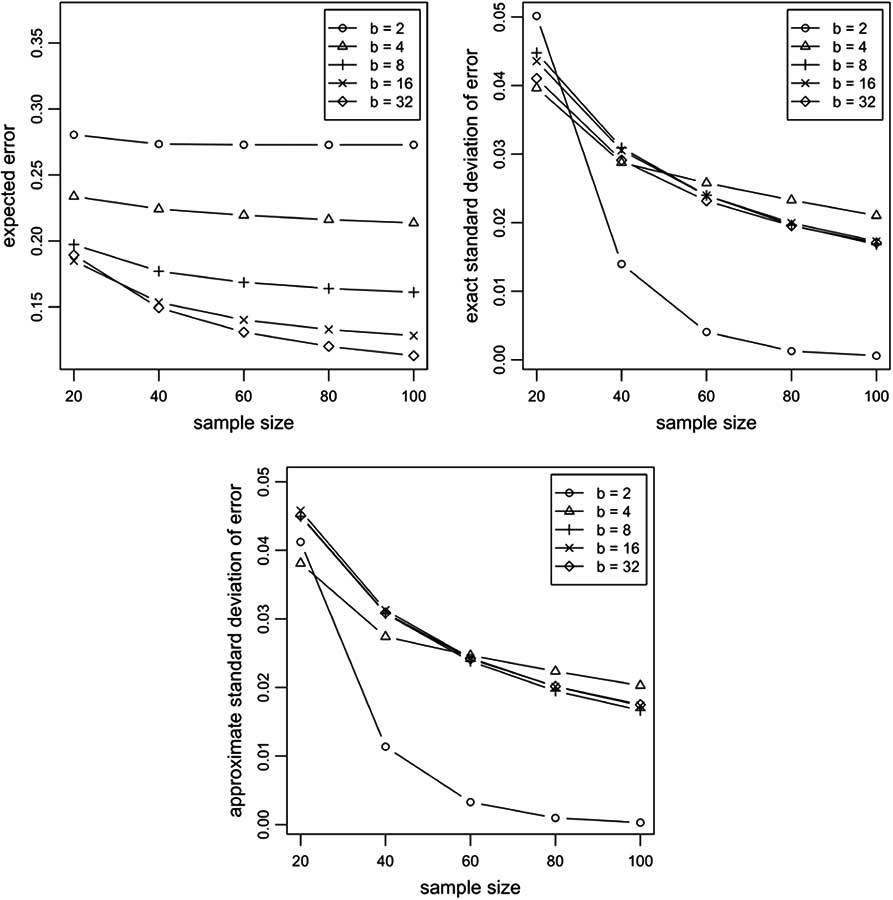
Performance of the discrete histogram classification rule.

**Fig. (4):**
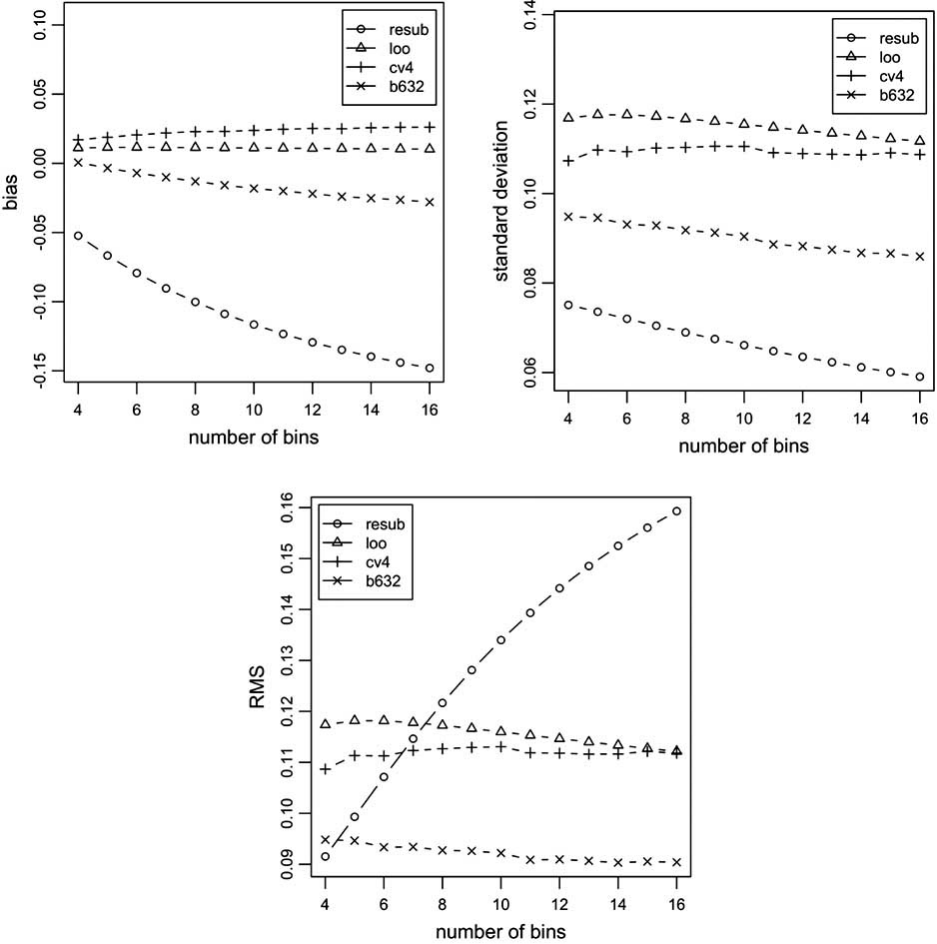
Performance of error estimators, for *n* = 20 and *E*[*ε_n_*] = 0.2. The values for resubstitution and leave-one-out are exact; the values for the other error estimators are approximations based on Monte-Carlo computation.

**Fig. (5):**
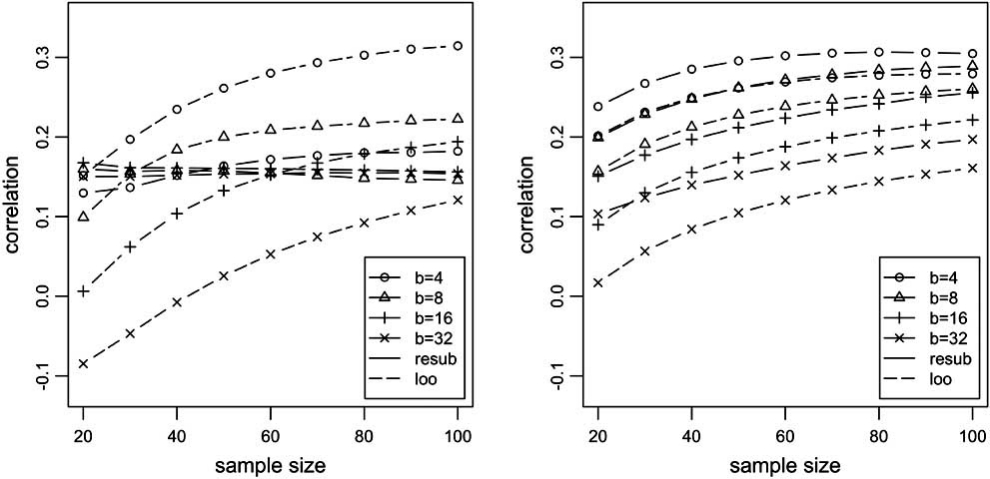
Exact correlation between the actual error and the resubstitution and leave-one-out cross-validation error estimators for probability models of distinct difficulty, as determined by the Bayes error. Left plot: Bayes error = 0.10. Right plot: Bayes error = 0.40.

**Fig. (6):**
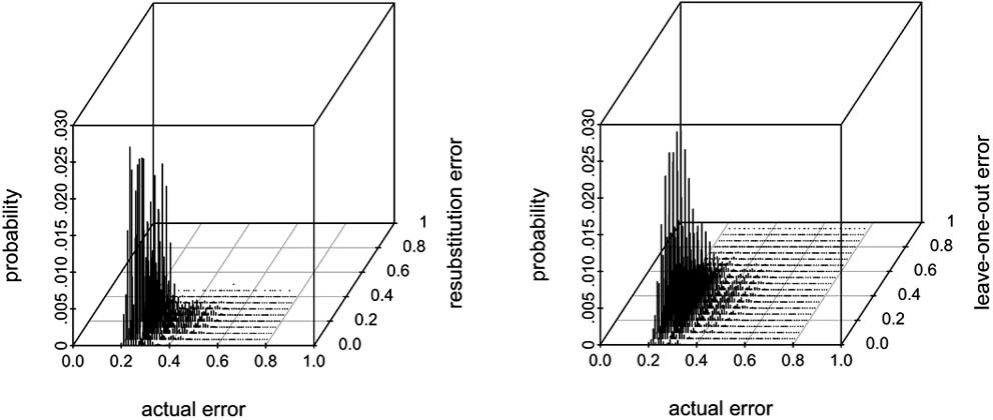
Exact joint distribution between the actual error and the resubstitution and leave-one-out cross-validation error estimators, for *n* = 20 and *b* = 8 , and a Zipf probability model of intermediate difficulty (Bayes error = 0.2).

**Fig. (7):**
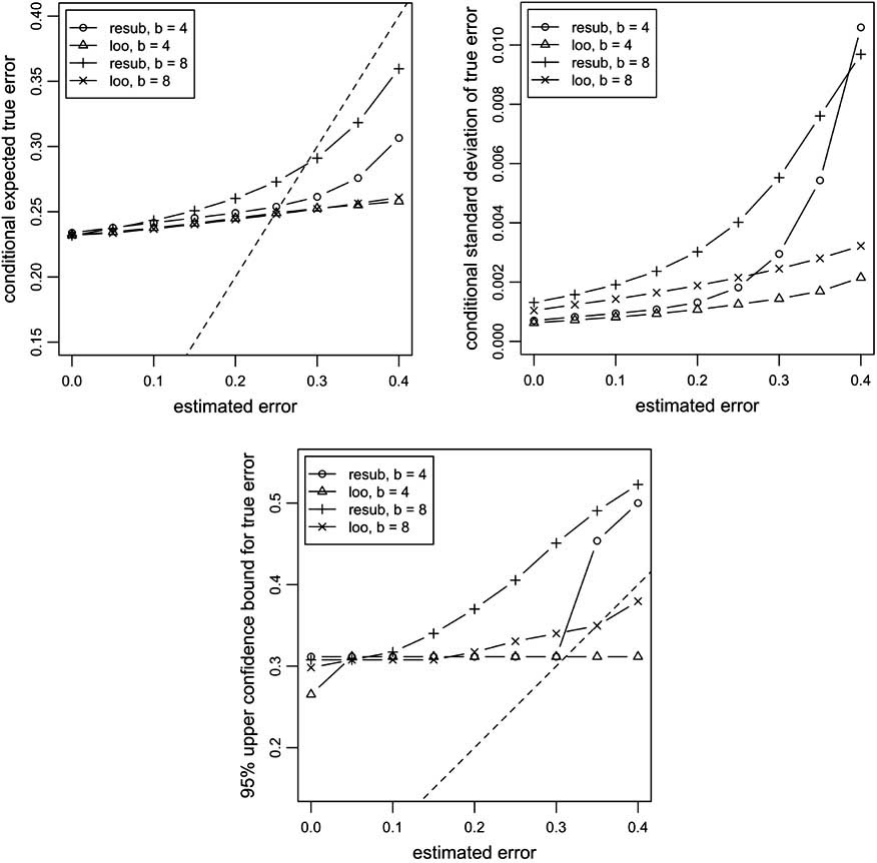
Exact conditional metrics of performance for resubstitution and leave-one-out error estimators. The dashed line indicates the *y = x* line.

**Fig. (8):**
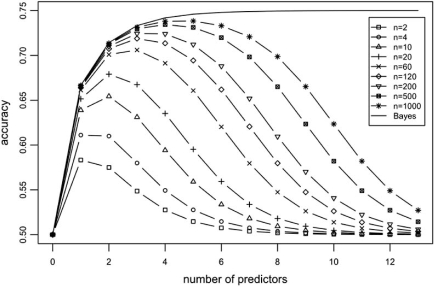
Average Bayes accuracy and average expected actual accuracy plotted as a function of number of binary predictors *p* = log_2_ (*b*) .

**Fig. (9):**
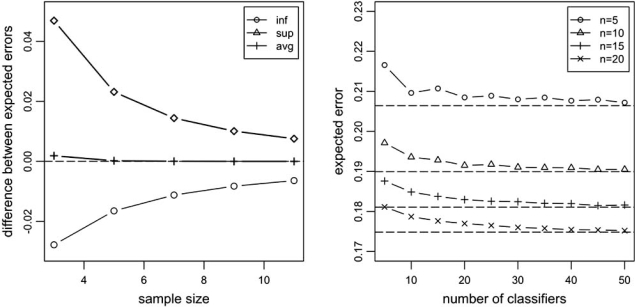
Performance of ensemble discrete classification rules, for *p* = 2 . Left plot: bounds and average difference between expected errors of the jackknife and discrete histogram classification rules, as a function of sample size, for *c_0_* = 0.5 . Right plot: expected error for the bagged discrete histogram classification rule, found by Monte-Carlo computation, as a function of number of classifiers in the ensemble, for model parameters derived from an actual data set. Also indicated are the exact expected errors of the base discrete histogram classification rule, by means of dashed horizontal lines, to which the expected error of the bagged classification rule in each case is clearly converging, as expected.
